# Catalytic Performance of One-Pot Synthesized Fe-MWW Layered Zeolites (MCM-22, MCM-36, and ITQ-2) in Selective Catalytic Reduction of Nitrogen Oxides with Ammonia

**DOI:** 10.3390/molecules27092983

**Published:** 2022-05-06

**Authors:** Agnieszka Szymaszek-Wawryca, Urbano Díaz, Bogdan Samojeden, Monika Motak

**Affiliations:** 1 Faculty of Energy and Fuels, AGH University of Science and Technology, al. A. Mickiewicza 30, 30-059 Kraków, Poland; bsamo1@agh.edu.pl (B.S.); motakm@agh.edu.pl (M.M.); 2 Instituto de Tecnología Química, Universitat Politècnica de València—Consejo Superior de Investigaciones Científicais, Avd. de los Naranjos s/n, 46022 Valencia, Spain; udiaz@itq.upv.es

**Keywords:** MWW zeolites, iron catalysts, one-pot synthesis, DeNO_x_

## Abstract

The application of layered zeolites of MWW topology in environmental catalysis has attracted growing attention in recent years; however, only a few studies have explored their performance in selective catalytic reduction with ammonia (NH_3_-SCR). Thus, our work describes, for the first time, the one-pot synthesis of Fe-modified NH_3_-SCR catalysts supported on MCM-22, MCM-36, and ITQ-2. The calculated chemical composition of the materials was Si/Al of 30 and 5 wt.% of Fe. The reported results indicated a correlation between the arrangement of MWW layers and the form of iron in the zeolitic structure. We have observed that one-pot synthesis resulted in high dispersion of Fe^3+^ sites, which significantly enhanced low-temperature activity and prevented N_2_O generation during the reaction. All of the investigated samples exhibited almost 100% NO conversion at 250 °C. The most satisfactory activity was exhibited by Fe-modified MCM-36, since 50% of NO reduction was obtained at 150 °C for this catalyst. This effect can be explained by the abundance of isolated Fe^3+^ species, which are active in low-temperature NH_3_-SCR. Additionally, SiO_2_ pillars present in MCM-36 provided an additional surface for the deposition of the active phase.

## 1. Introduction

Zeolites belong to a large group of materials that found widespread application in industrial catalytic reactions [1,2]. The high potential of those aluminosilicates is ascribed to their frameworks with uniform pores of molecular dimensions [3]. Recently, the area of layered zeolites belonging to the MWW (Mobil tWnety-tWo) family has gained growing attention due to their unique structural parameters [4,5]. The common feature of these materials is a crystalline unit, the so-called MWW monolayer. This structure contains a 2D 10-member ring (MR) sinusoidal channel pore system of 25 Å thickness, cups of 7.1 Å dimension, and a depth of 7.0 Å. The representatives of this group are MCM-22, MCM-36, and ITQ-2 [6,7,8]. The materials are derived from the same layered precursor, MCM-22 (P), obtained by hydrothermal treatment of aluminosilicate gel containing hexamethyleneimine (HMI) as a structure-directing agent. The precursor contains a two-dimensional structure, with the sheets electrostatically bonded by weak Van der Waals forces. Thus, the material can be relatively easily transformed into condensed, disorganized, or delaminated zeolite [6]. The basic procedure to modify the precursor is calcination, which yields a three-dimensional crystal framework of MCM-22 with two independent pore systems [9]. The alternative procedure of modification is intercalation with inorganic or/and organic pillars, introduced into the interlayer space of MCM-22 (P). In the case of MCM-36, the first step of the pillaring procedure is swelling of the precursor with organic surfactant. Subsequently, the swollen material is pillared with SiO_2_, using tetraethoxysilane (TEOS) as a precursor. MCM-36 obtained after hydrolysis and calcination exhibits microporosity in crystalline layers and mesoporosity in the interlayer region. Consequently, the access to the internal space of MCM-36 is facilitated, and additional active sites can be easily introduced between the zeolitic sheets [10]. Another material obtained from MCM-22 (P) is ITQ-2. It is characterized by homogeneous external surface area of around 700 m^2^·g^−1^ and a structure formed by single MWW layers of 25 Å thickness, organized in a “house of cards” arrangement [6,11]. ITQ-2 was synthesized and described for the first time by Corma and co-workers [12]. The authors emphasized that the zeolite exhibits significantly higher amounts of structurally accessible acid sites than MCM-22 or MCM-36.

Many studies investigated the catalytic potential of the layered zeolites of the MWW family in their pristine and metal-modified form. Wang et al. [13] claimed that dealuminated MCM-22 exhibited significantly higher activity and selectivity in the catalytic cracking of *n*-hexane to propylene than H-ZSM-5 or H-Beta zeolites. Moreover, the work by dos Santos [14] suggested that Fe^3+^-exchanged MCM-22 showed a very good catalytic performance in the production of acrylic acid in the reaction of oxidative dehydration of glycerol. Apart from the organic reactions, modified MCM-22 can also be used as a catalyst for the processes that include inorganic reactants. For instance, Rutkowska et al. [15] proposed Fe-modified MCM-22 as a very efficient catalyst for N_2_O decomposition. Moreover, Chen et al. [16] carried out an experiment to find the most appropriate procedure to prepare Fe-MCM-22 catalyst for selective catalytic reduction of nitrogen oxides with ammonia (NH_3_-SCR). Moreover, modified ITQ-2 was tested in passive adsorption of NO_x_ [17] and initially, in NH_3_-SCR [5].

As it can be noticed, zeolites, including those of the MWW family, found an application in many environmental catalytic processes. One of the most meaningful problems of modern industry is the emission of nitrogen oxides (NO_x_), responsible for acid rain and photochemical smog formation or ozone layer depletion [18,19,20]. The most widespread technology to abate the emission of NO_x_ is NH_3_-SCR; however, due to some difficulties with the commercial vanadium-based catalyst, alternative systems have been extensively studied in recent years [21,22,23]. It is well-known that transition metal-modified zeolites are among the most promising substitutive catalysts of NH_3_-SCR. The most advantageous features of these materials are broad temperature window, relatively low emission of N_2_O during the reaction, resistance to the poisoning compounds, and well-defined pore structures [24,25,26,27]. There is a general agreement that zeolites modified with copper or iron exhibit the most satisfactory catalytic performance in NH_3_-SCR [25,28,29]. One important difference between the materials is their temperature window. Typically, Cu-zeolites show high NO conversion below 350 °C, while Fe-zeolites are the most active above this temperature. Such behavior of the catalysts is assigned to a different ability of reduction of Cu and Fe species present in the zeolitic frameworks [16,28]. What is more, predominantly lower reduction of NO obtained by Cu-exchanged zeolites is caused by the parallel, undesired oxidation of ammonia [29]; thus, consumption of the reducing agent. In contrast, iron-modified materials offer significantly higher selectivity to N_2_ and negligible formation of N_2_O in a wide temperature region of NH_3_-SCR [5]. In the majority of NO_x_ emission sources, such as power plants, chemical industry, or gas turbines, the operational temperature is relatively high [30]; therefore, the continuation of the studies on Fe-modified zeolites as new NH_3_-SCR catalysts is highly required. What is more, aluminosilicates modified with Fe were confirmed to exhibit high reusability in many catalytic processes, which is highly required for the materials used on the industrial scale [31].

According to the literature, the catalytic behavior of Fe-containing zeolites in NH_3_-SCR is determined by the speciation of iron [32,33,34]. It was proved that the final form of Fe sites depends on the modification method, and many scientists attempted to optimize their distribution in zeolitic frameworks [16,34,35,36]. Brandenberger and co-workers [34] provided a detailed investigation of the dependency of the iron active site activity in Fe-ZSM-5 on the reaction temperature. The research showed that satisfactory performance of the catalyst below 300 °C was a consequence of the presence of isolated iron cations. Instead, with the increasing temperature, dimeric and oligomeric species had a higher contribution to NO reduction. The authors introduced Fe into the ZSM-5 framework by the ion-exchange procedure; however, despite the satisfactory distribution of the active phase, there are some obstacles to the application of this method. Usually, it is necessary to perform multiple ion-exchange cycles in order to reach a satisfactory content of the active phase. Additionally, it is difficult to determine the exact amount of metal introduced into the support on the synthesis level. As an alternative to ion exchange, the active phase can be introduced by incipient wetness impregnation. In this case, the percentage of the metallic forms can be introduced with high precision. Nevertheless, impregnation with metal salts causes the formation of metal oxide clusters or bulk particles, which drastically decreases the specific surface area and hinders access to the isolated active centers. Specifically, in the case of NH_3_-SCR, the aggregated species can cause side reactions [34].

Taking into consideration that the distribution of iron plays an essential role in catalytic performance, the application of the appropriate modification procedure is crucial to obtaining satisfactory NO conversion. The above-mentioned limitations of ion exchange and incipient wetness impregnation (classified as common post-synthesis modifications) can be eliminated by choice of direct hydrothermal synthesis of Fe-zeolites, called “one-pot synthesis” (OPS). This procedure offers both a reduction in the preparation steps and allows obtaining a catalyst with a homogeneous distribution of metallic, active sites [37]. The efficiency of the preparation of Fe-zeolites in various frameworks by the OPS procedure has been investigated by many researchers [16,28,35,37,38,39]. Nevertheless, to the best of our knowledge, only one study has examined the properties of one-pot synthesized Fe-MWW zeolite, using MCM-22 as the exemplary catalyst support [16]. It was declared that, in comparison to Fe-MCM-22 modified by post-synthesis methods, the OPS-synthesized catalyst showed enhanced catalytic activity, especially in low-temperature NH_3_-SCR.

The highly promising catalytic performance of one-pot synthesized Fe-MCM-22 in NH_3_-SCR was a motivation for us to investigate whether other layered zeolites of the MWW family, MCM-36 and ITQ-2 with Fe, can be successfully prepared by OPS procedure; therefore, our study aimed to determine the impact of the addition of iron precursor into the synthesis pot on the speciation of the active phase in the zeolitic frameworks and catalytic performance of the obtained materials in NH_3_-SCR.

## 2. Results and Discussion

### 2.1. Physico-Chemical Properties of the Materials

#### 2.1.1. Chemical Composition and Crystal Structure

The chemical composition of the pristine zeolites and the catalysts with regard to Si, Al, and Fe content was measured using ICP-AES. The obtained results are collected in Table 1. Since all of the materials in our study were prepared from the same synthesis gel, the amount of Si and Al in the pure zeolites is very similar. Negligible differences in the detected amounts that directly affect the Si/Al molar ratio can be caused by the apparatus error. Additionally, no significant difference was found between the content of iron in Fe-MCM-22, Fe-MCM-36, and Fe-ITQ-2; however, from the data shown in Table 1, it can be noted that Si/Fe molar ratio is slightly lower compared to the expected, calculated value.

**Figure 1:**
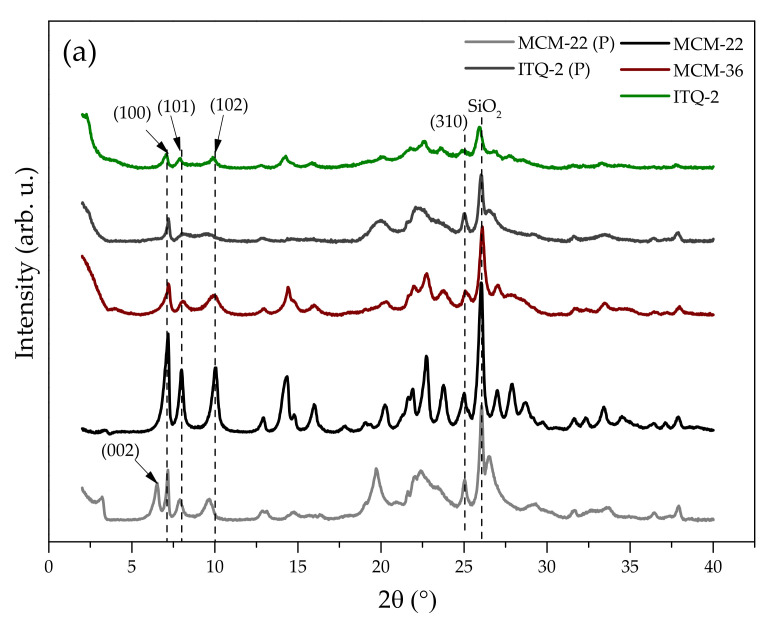

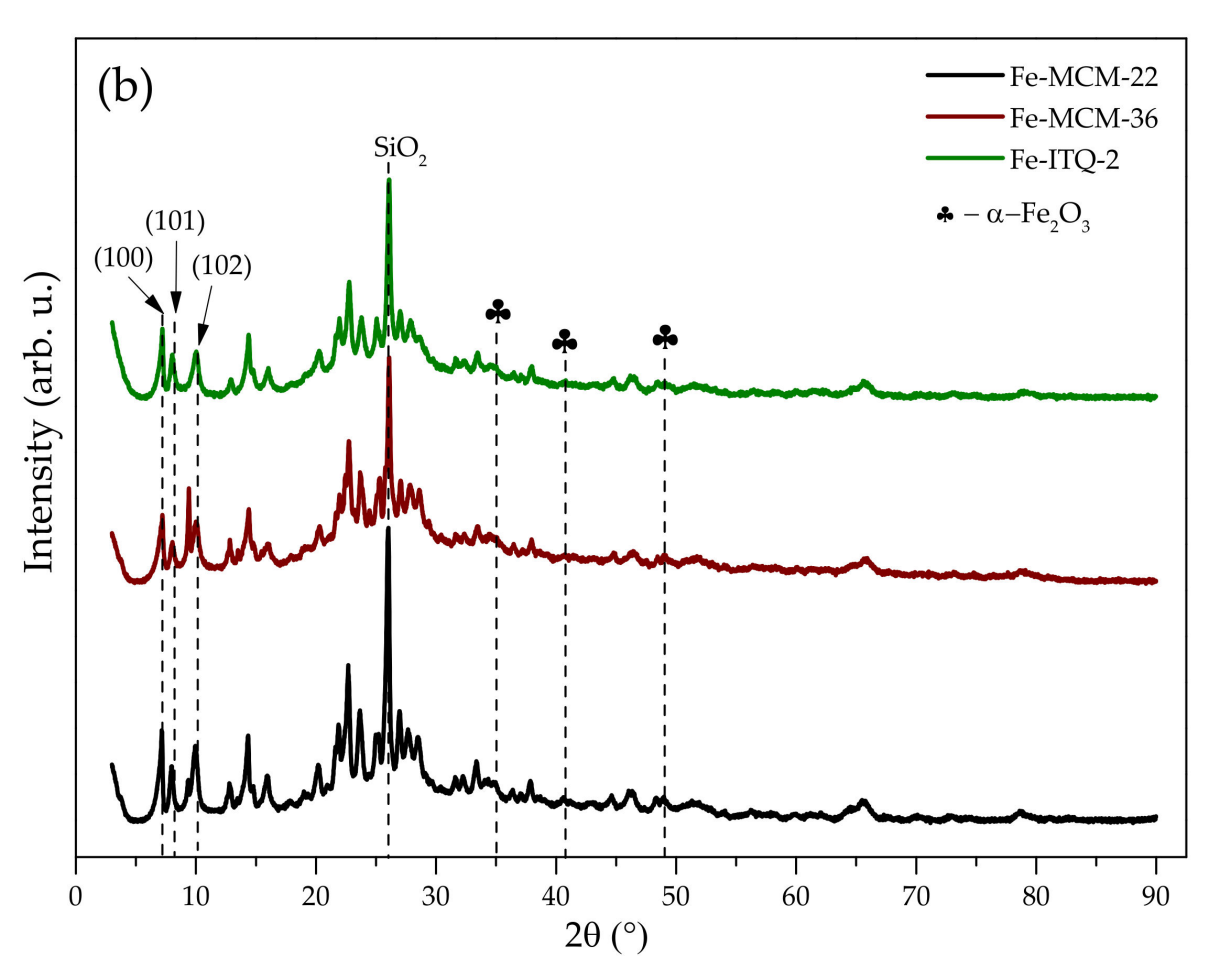
XRD patterns of the investigated materials: (**a**) MCM-22 (P) and its pristine derivatives in *2θ* range of 2–40°; (**b**) iron-modified layered zeolites in *2θ* range of 3–90°.

X-ray diffraction (XRD) analysis was conducted to recognize the characteristic structure of the materials and the distance between the layers. The patterns in the *2θ* range of 2–40° and 3–90° are presented in Figure 1a,b, respectively. Figure 1a reveals a relationship between the modifications of MCM-22 (P) and the crystal structure of its derivatives. The diffraction maxima in the *2θ* range of 6–10 ° enable us to indicate the layers within the zeolitic framework [40]. The presence of so-called diagnostic reflections with their (*hkl*) indices at *2θ* of 6.6° (002), 7.1° (100), 8.0° (102), 9.8° (220), 25° (310), and 26° proves that the performed synthesis yielded highly crystalline layered precursor. Additionally, the formation of the layered structure is confirmed by the appearance of the reflection at 3.1° in the XRD pattern of MCM-22 (P) [40]. Another strong evidence of the formation of MCM-22 (P) is the presence of the distinct doublet at 6.5–7.1°. The observation is significant due to the possibility of the generation of MCM-49 during the synthesis, which is typically proved by the presence of a diffraction peak at 7.1° [41] The interlayer (002) diffraction maxima at 6.6° is assigned to the ordered layer structure [42]. Since it corresponds to *d*-spacing of 1.3 nm, two adjacent and perpendicularly ordered MWW layers are separated from each other by 2.6 nm.

Calcination of MCM-22 (P) resulted in the removal of the template molecules from the interlayer space. As was confirmed by He et al. [43], calcination of the layered precursor results in the connection of the hexagonal sheets upon irreversible condensation of OH groups and the formation of the second pore system. As a result, (002) reflection overlapped with the intra-layer reflection (100), indicating the formation of a three-dimensional microporous structure [44]. Moreover, the calcination procedure resulted in an increase in the intensity of the intra-layer (100) and inter-layer mixed (101) and (220) diffraction maxima present in the *2θ* range of 12–25°. The XRD lines became sharper and well-resolved; however, their position remained unchanged, which proves satisfactory crystallinity and phase purity of MCM-22.

Taking into account the irreversible condensation of hydroxide groups during calcination, MCM-36 is prepared from a “wet cake” of MCM-22 (P). In contrast to MCM-22, the atomic order and layer registry in the third dimension along the *c* axis disappeared in the case of MCM-36. This can be confirmed by the presence of the broad band in the *2θ* range of 8–10°. Hence, the material exhibits a two-dimensional MWW topology and partially delaminated structure [7]. In addition, the characteristic reflection at (002) of the precursor disappeared, with the simultaneous appearance of highly intense low-angle diffraction maximum present at *2θ* ca. 2°. This reflection corresponds to a *d*-spacing of around 4.15 nm. Considering that the thickness of the MWW layer equals approximately 2.5 nm [45], the results evidenced successful intercalation of the material with amorphous SiO_2_ pillars and the separation of the layers by ca. 1.65 nm.

Delamination and subsequent calcination of the swollen MCM-22 (P) yields ITQ-2 with the characteristic “house of cards” structure [46]. As can be seen in the XRD pattern of ITQ-2 (P), the sample showed an intense diffraction maximum below 2°, which indicates the increase in the interlayer distance due to the introduction of the surfactant molecules. Calcination resulted in the disappearance of the above-mentioned maximum, together with that at (002). Moreover, the sharp reflections at *2θ* of ca. 7.1° (100) and 25° (310) became broader, suggesting a reduction in the long-range structure order. Hence, the precursor was successively delaminated and the layers of the resulting product were randomly oriented [7,47]. Full delamination of the precursor is also confirmed by the lower intensity of the reflections of ITQ-2, compared to MCM-36 [48].

The influence of Fe addition into the synthesis pot on the crystal structure of the zeolites was analyzed in the *2θ* range of 3–90°. The presence of iron oxide particles in the zeolitic framework was also determined in this region. It can be noted from Figure 1b that the addition of iron did not change the position of the characteristic structural reflections; therefore, the ordered, pillared, or disorganized arrangement of the layers was not affected by the substitution of Si^4+^ or Al^3+^ with Fe^3+^ cations. The presence of diffraction maxima at *2θ* of 35.5°, 40.7°, and 49° of very low intensity can be ascribed to the formation of small particles of α-Fe_2_O_3_ [10,49]. The result suggests that iron was not only incorporated into the aluminosilicate framework but also deposited in an oxide form. This indication is in line with UV-Vis analysis, which demonstrated diversified speciation of Fe in the catalysts.

#### 2.1.2. Textural Properties

**Figure 2:**
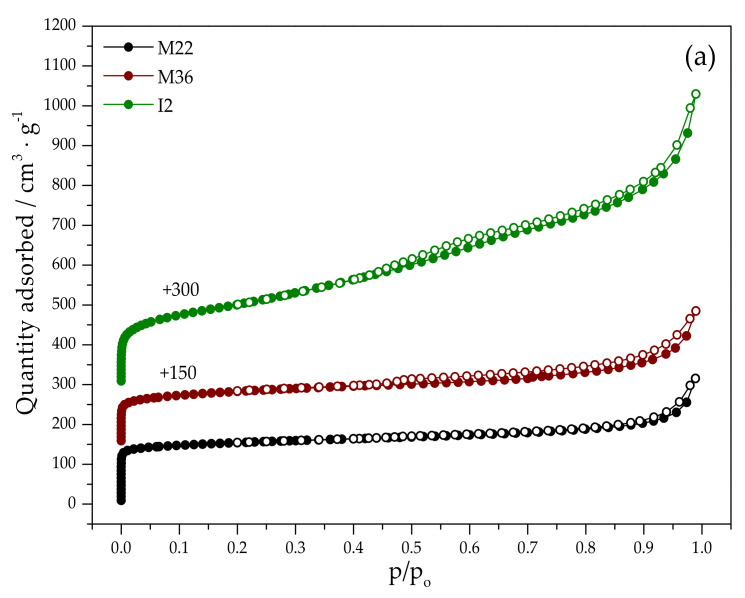

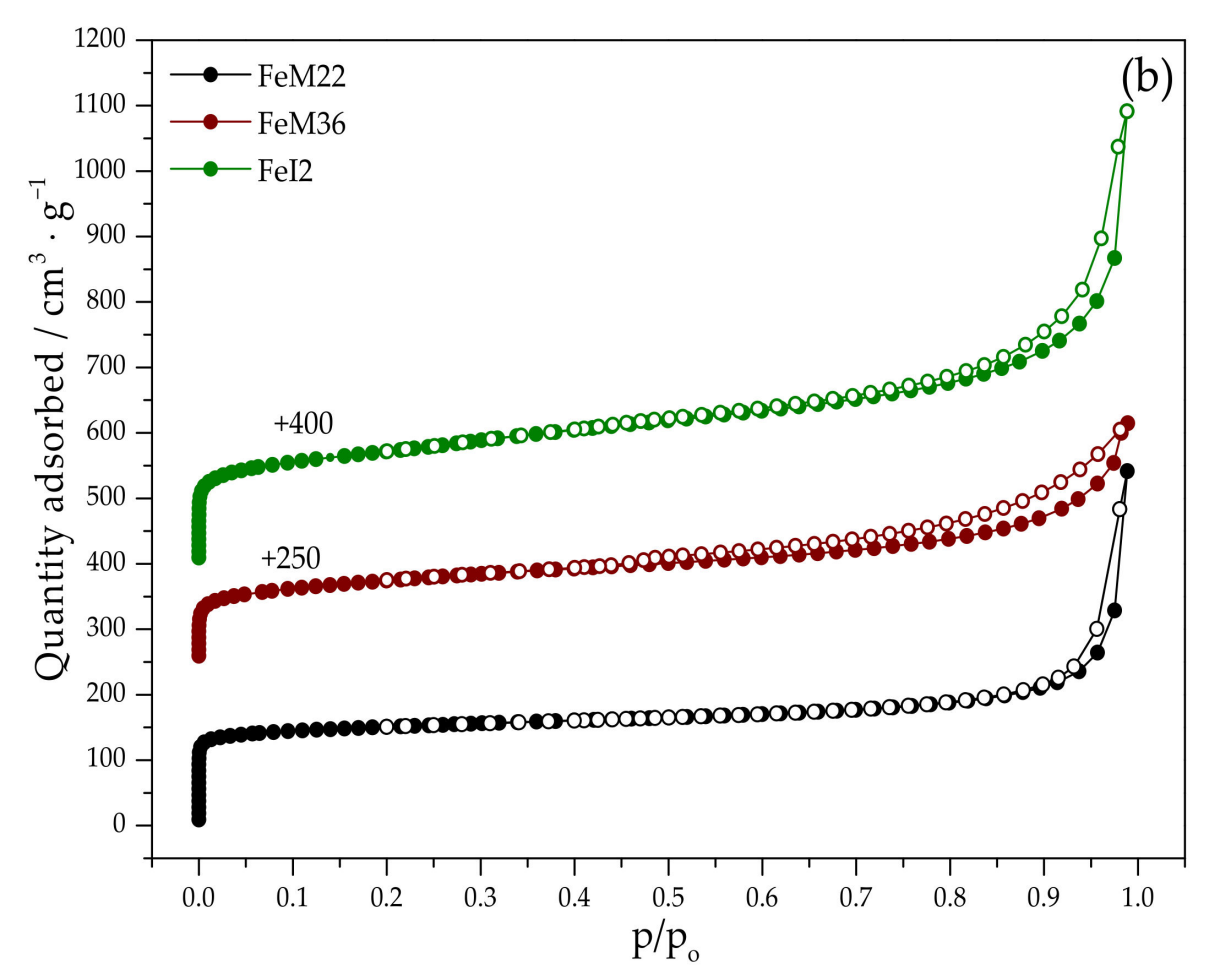
N_2_ sorption isotherms obtained for the investigated materials: (**a**) pristine MWW zeolites; (**b**) iron-modified MWW zeolites.

Nitrogen adsorption–desorption measurements were performed to identify distinct textural properties correlated with the diversified porosity of the materials. The resulting isotherms are presented in Figure 2, while textural and structural parameters of the samples are collected in Table 2. One of the typical features of MWW materials is the presence of the hysteresis loop, which was observed for all the analyzed samples; therefore, all of them are comprised of variously packed layers [43].

In the case of M22, the shape of the N_2_ adsorption branch is characteristic of type I (b) of the IUPAC classification [50]. The isotherm exhibits a sudden increase in N_2_ adsorption at a very low value of p/p_0_, which is assigned to the microporous structure of the material [10,46]. It can be observed that even at p/p_0_ of 0.1, the majority of the sorption capacity is used due to the strong adsorption driving force of micropores; however, the characteristic shape and the hysteresis loop of type H4 suggest a broader range of pore size distribution. Thus, the generation of secondary mesopores formed by non-rigid aggregates of plate-like particles and slit-shaped pores is possible for this material. Modification with iron resulted in the transformation of the isotherm type into IV (a)-like and the hysteresis loop into type H3. Type IV (a) of the adsorption branch is given usually by mesoporous materials, in which capillary condensation takes place [51]. Nevertheless, the isotherm did not exhibit the characteristic final saturation plateau. Thus, after modification with Fe, the zeolite became more mesoporous, but still preserved microporosity. The speculation is in line with the data presented in Table 2, since the participation of meso- and macroporosity increased from 60% for M22 to 64% for FeM22.

The adsorption branches recorded for M36 exhibited a similar profile to M22; however, nitrogen condensation at low p/p_0_ was slightly lower for the pillared zeolite, indicating the presence of mesoporosity. A considerable volume of mesopores can also be explained by the increased adsorption capacity above p/p_0_ (0.4) and noticeably larger H4 hysteresis loop compared to M22 [10,12]. This type of isotherm is assigned to slit-like pores or platy particles [49]. According to He et al. [43], mesopores in MCM-36 are created along two routes during the calcination procedure. On the one hand, the polymeric silicon hydroxide forms long polymeric chains between two adjacent layers and distances them from each other. Alternatively, the molecules of the organic compounds of the swelling solution are removed, leaving empty holes within the aluminosilicate framework; therefore, the presence of SiO_2_ pillars resulted in a lower volume of nitrogen adsorbed at low p/p_0_ compared to M22 or I2. After modification with iron, both the isotherm and the hysteresis loop reflected the same shape as M36; however, the results collected in Table 2 highlight that, similar to FeM22, FeM36 contains a higher amount of mesopores compared to the pristine zeolite. Interestingly, the introduction of iron only slightly changed the surface or the volume of micropores. This effect can be related to the isomorphous incorporation of Fe^3+^ into the zeolitic framework or the deposition of metallic oligomers within the openings of mesopores.

The pure delaminated zeolite, I2 exhibited its characteristic isotherm shape [42]. The adsorption–desorption branch was a mixture of I (a) and IV (a) types [51]; therefore, despite delamination, the material still contains a peripherally microporous structure; however, a stepwise increase in the adsorption capacity above p/p_0_ 0.5 suggests the presence of well-developed mesopores. The hysteresis loop of I2 is type H3, which is characteristic of plate-like particles [51]. It can be observed that the incorporation of iron into the zeolitic framework noticeably influenced the shape of the adsorption branch. In the p/p_0_ range of 0.5–0.7, the adsorption–desorption lines are almost identical and slightly inclined, while for p/p_0_ of 0.7–1.0, the hysteresis loop resembles that of FeM22; therefore, iron in the synthesis pot facilitated the formation of more regular mesopores, similar to FeM22. Additionally, the decrease in the specific surface area in the case of this sample after modification with iron was the highest among all of the materials.

Taken together, the obtained results suggest that the specific surface area of the pristine or iron-modified MWW zeolites increased in the following order: (Fe)M36 < (Fe)M22 < (Fe)I2. On the other hand, the growth of the pore volume was (Fe)M22 < (Fe)M36 < (Fe)I2; however, the difference in this value for (Fe)M22 and (Fe)M36 is negligible. The reported results are in agreement with the literature studies [5,12,44]. Additionally, its microporosity increased after the introduction of iron into zeolites. Moreover, the presence of the characteristic “point B” in the isotherms suggests micropore filling, while the hysteresis loop proves the presence of the mesoporous structure of the materials.

#### 2.1.3. Acidity of the Catalysts

**Figure 3:**
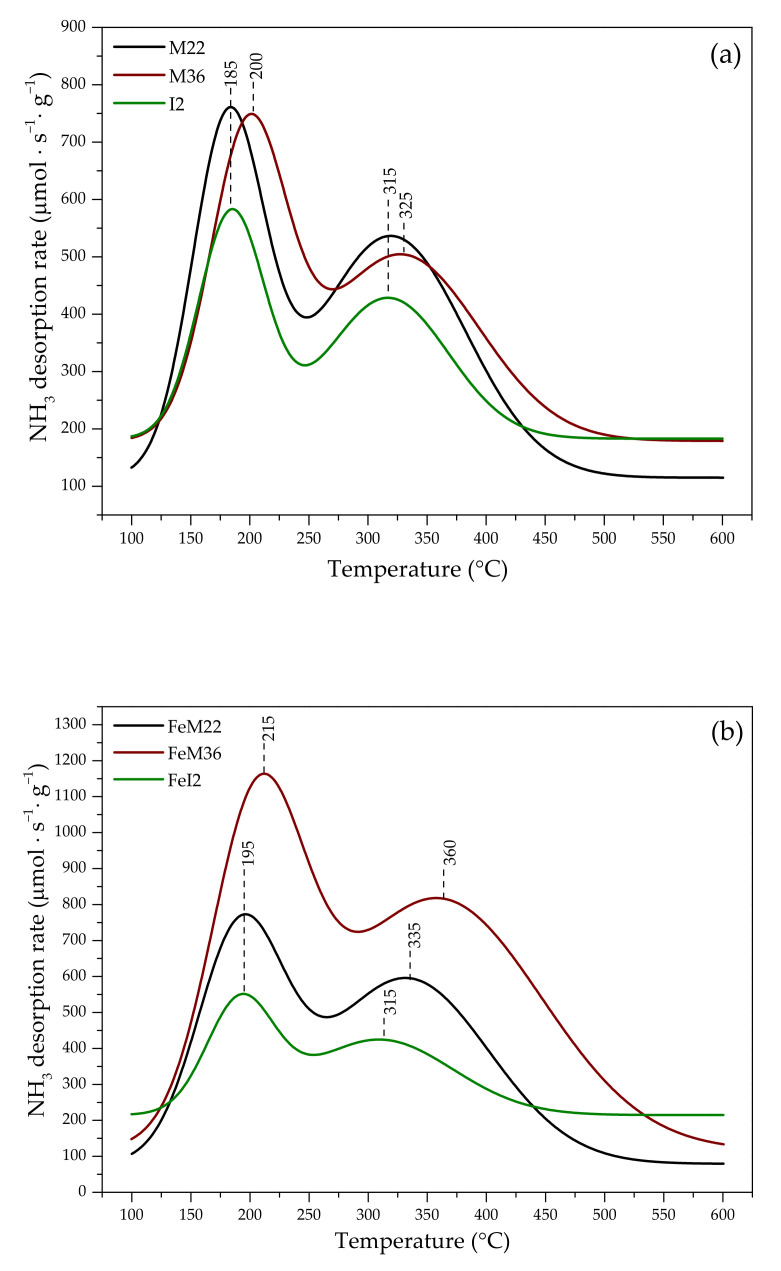
NH_3_-TPD profiles obtained for (**a**) pristine MWW zeolites; (**b**) iron-modified MWW zeolites.

In order to identify the quantity, distribution, and strength of the acidic sites of the pristine and Fe-modified zeolites, the samples were subjected to temperature-programmed ammonia desorption (NH_3_-TPD) experiments. NH_3_-TPD profiles obtained for the pure and Fe-modified zeolites are presented in Figure 3. The quantitative evaluation of the number of weak and strong acid sites is presented in Table 3. As can be seen in Figure 3, the obtained profiles can be divided into two regions, ascribed to the desorption of ammonia from the sites of low (low-temperature peak, LT) and strong (high-temperature peak, HT) strength, respectively [52]. The peaks observed below 250 °C result from the desorption of weakly adsorbed NH_3_ molecules on Lewis sites, while the peaks above this temperature appear due to the removal of ammonia from the Brönsted sites [53]. It can be observed from Figure 3a that the distribution of the acidic sites strongly depends on the pillaring or delamination of MWW zeolites. M22 exhibits the abundance of the acid centers of low strength, which desorbed ammonia molecules at 185 °C, and a significantly lower amount of the strong acid sites, desorbing NH_3_ at 315 °C. Interestingly, the position of desorption peaks of I2 corresponds to that of M22; however, their intensity is significantly lower, which is in line with studies found in the literature [54,55,56]. The lower acidity of I2, compared to M22, can be ascribed to the partial dealumination of ITQ-2 during swelling, the application of a highly acidic environment, and hydrolysis of the Al–O–Si bonds during the synthesis [54]. For M36, both of the desorption peaks are slightly shifted to the higher temperature region of 200 and 325 °C, respectively. The concentration of the acidic sites of higher strength is lower in comparison to M22 but noticeably higher than that of I2. The effect results from the introduction of non-acidic silica pillars that increase the mass of the sample but do not contribute to the formation of new acidic centers. Moreover, SiO_2_ could partially block the sites for NH_3_ adsorption [57]. According to the data presented in Table 3, the total acidity of MWW zeolites can be ordered as M22 > M36 > I2, which corresponds to the previously reported studies [5,57].

As shown in Figure 3b, the introduction of iron into the synthesis pot had a considerable influence on the formation of acidic centers. The significant increase in NH_3_ adsorption capacity can be explained by the fact that one Fe^3+^ is able to adsorb two molecules of ammonia [58]. Additionally, there is a correlation between the organization of the layers of Fe-modified materials and their total acidity. In the case of M22 and M36, the total amount of desorbed NH_3_ molecules increased after the introduction of iron, while for FeI2, it declined. Additionally, the desorption temperature from both weak and strong centers increased to 195 °C and 335 °C for FeM22 and to 215 °C and 360 °C for FeM36. Thus, iron promoted not only the formation but also the strength of the newly generated acidic centers. For FeI2, the temperature of ammonia desorption from weak sites moderately increased to 195 °C, but the strong sites remained unchanged compared to the pristine support. This effect suggests that Fe^3+^ cations incorporated into the zeolitic framework generated stronger bonds with NH_3_ molecules than Al^3+^ sites. Surprisingly, after the introduction of Fe into the synthesis pot, FeM36 exhibited the highest total concentration of the acidic centers among all the iron-modified samples. This effect can be associated with the presence of SiO_2_ pillars, which provide additional surfaces for the deposition of iron species. Alternatively, the presence of iron could result in the partial replacement of (≡Si−O(H)−Al≡) by Fe^3+^ cations, and thus, the transformation of Brönsted acid sites into Lewis acid sites [10]. What is interesting, our conclusions do not follow those of Jankowska and co-workers [10], who reported that the deposition of iron decreased the surface concentration of acidic sites in MCM-22 and MCM-36. The authors claimed that the formation of aggregated iron oxide species could limit the diffusion of ammonia molecules to the acidic centers due to pore clogging. Nevertheless, one should note that the authors deposited Fe sites by the ion-exchange procedure, which could result in the aggregation of iron oxide particles. Hence, one-pot synthesis of Fe-MWW-catalysts is much more advantageous for the generation of new acid centers compared to ion exchange. Our explanation corresponds to the outcomes of UV-Vis, which proved the abundance of well-dispersed monomeric Fe^3+^ cations in FeM36, contributing to Lewis-type acidity. Furthermore, the binuclear [HO−Fe^3+^–O–Fe^3+^–OH]^2+^ sites created stronger bondings with NH_3_ molecules, which are broken at higher temperatures. The presence of the isolated and binuclear iron moieties explains the increased acidity of FeM22 as well; therefore, our results are in agreement with the fact that the additional Fe^3+^ species in zeolites can form new acid sites in the framework [59]. Moreover, the lowest acidity of FeI2 can result from its delaminated structure and deposition of more aggregated Fe_x_O_y_ clusters. These species could partially block the acidic centers of the material and did not deliver any new centers for NH_3_ adsorption. Additionally, the possible dealumination of the sample during the synthesis could contribute to the loss of Brönsted acidity, provided by Al–O–Si moieties [55].

#### 2.1.4. Characteristic Chemical Groups Present in the Materials

**Figure 4:**
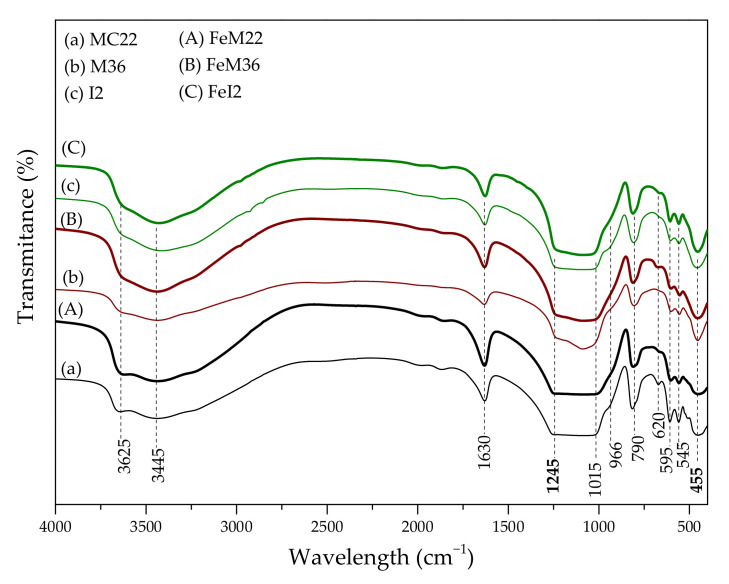
FT-IR spectra of the pristine and Fe-modified MWW zeolites.

The characteristic functional groups in the pristine and Fe-modified materials were studied using FT-IR spectroscopy. The obtained spectra, presented in Figure 4, exhibited a shape typical for MWW layered materials. It is apparent that the modification with iron did not induce any changes in the zeolitic frameworks. In general, the presence of aluminosilicate structures is confirmed by the peaks present in the spectra in the region of 1300–400 cm^−1^ [60]. The peak at 455 cm^−1^ can be ascribed to M–O bending vibrations (where M = Si and Al) [10]. The confirmation of the double-six-ring (D6R) MWW topology in all of the materials can be found at 595 cm^−1^ and 545 cm^−1^ [44,60]. Since the bands are detected for all the tested samples, the introduction of iron did not interrupt the formation of the zeolitic frameworks. Furthermore, the peak at 620 cm^−1^ highlights the presence of out-of-plane coupled vibrations of Si–O and Al–O bonds, while the one at 790 cm^−1^ can be ascribed to the stretching vibrations of SiO_4_^2−^ tetrahedra [61]. The shape of another characteristic band at 1015 cm^−1^, assigned to the asymmetric internal vibrations of the zeolitic framework, changed after the introduction of iron. Thus, metal cations were successfully incorporated in place of silicon and/or aluminum cations. Other absorption bands, located at 966 cm^−1^ and 1245 cm^−1^, are related to the silanol groups and the stretching modes of M–O–Si, (where M = Al or Fe), respectively [62]. Interestingly, for I2, the peak at 966 cm^−1^ is better resolved and more intense than for M22 and M36, due to the abundance of the corresponding groups on the external surface area of the delaminated zeolite. The intense peak at 1630 cm^−1^, present for all samples, appears due to the physically bonded water molecule [63]. The bands detected within 4000–3000 cm^−1^ are assigned to hydroxyls attached to the framework: the broad one at 3445 cm^−1^ is attributed to hydrogen–oxygen bonds in OH groups [64], while the sharper one at 3625 cm^−1^ is characteristic for Brönsted acidic sites of Si(OH)Al in the supercages at the 10 member-ring channels [65,66]. Since the latter peak is more intense for Fe-zeolites, it can be assumed that the introduction of metal resulted in the formation of new acidic centers within the aluminosilicate structure. The important observation is that the peak at 3625 cm^−1^ is the most intense for M22. This result can be related to the difference in the structure of this zeolite compared to M36 and I2.

#### 2.1.5. Speciation of Iron

**Figure 5:**
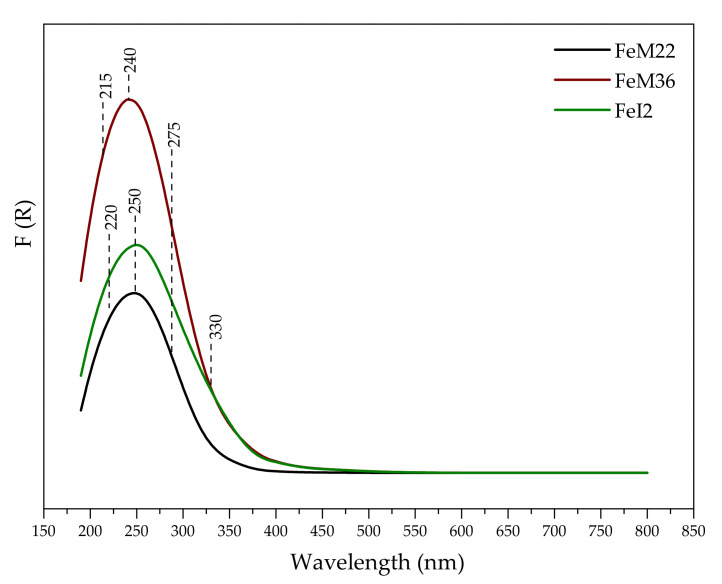
UV-Vis spectra obtained for Fe-MWW layered zeolites.

Speciation of iron introduced into the zeolitic frameworks was investigated using UV-Vis spectroscopy. The obtained absorbance spectra are presented in Figure 5. In general, in the case of Fe-containing materials, three characteristic regions can be distinguished: 200–350 nm, arising from O → Fe^3+^ charge transfer of isolated Fe^3+^ cations in the framework or extra framework positions; 350–450 nm, corresponding to octahedral Fe^3+^ moieties in small oligomers of extra framework Fe_x_O_y_ clusters; above 450 nm, assigned to larger and more aggregated bulks of hematite (Fe_2_O_3_) [67]. It can be noted from Figure 5 that the line shape of the spectra is similar for all of the catalysts; however, a significant difference was found in the intensity and positions of the bands. All of the samples exhibited a strong absorption band between 200 and 350 nm, with the maximum at 240 nm (FeM36) and 250 nm (FeM22 and FeI2). Moreover, the intensity of the adsorption line of FeM36 at 213 nm slightly increased. All of the bands are ascribed to the ligand (oxygen)-to-metal charge transfer (CT) transitions of mononuclear Fe^3+^ cations. According to the literature, their position depends on the number of ligands [68]. Bordiga et al. [69] confirmed that the presence of the absorption line at 215 and 240 nm is related to the isomorphous substitution of Si^4+^ by Fe^3+^ in the zeolitic framework. Our observations corroborate those obtained by Yang et al. [70] and Testa et al. [71], who reported domination of the Fe^3+^ framework monomers in one-pot synthesized FeM22. In the case of FeM22 and FeI2, the absorption lines are shifted to 220 and 250 nm, respectively. These bands are assigned to extra framework Fe^3+^ species in tetrahedral and octahedral coordination, respectively [72]. Thus, the isomorphous substitution in the zeolitic framework was not as effective, as in the case of FeM36. Moreover, the band at 275 nm, present in the case of all of the materials, suggests that iron monomers also appeared in octahedral coordination [73]. Additionally, the presented results showed a significant relationship between the zeolite structure and the form of the active phase. The intensive band at 330 nm, detected for FeM36 and FeI2 and absent for FeM22, confirmed the presence of oligonuclear clusters (Fe_x_^3+^O_y_) in extra framework positions [73]. The results reported by Gurgul et al. [74] indicated that the formation of the particular iron species depends on the metal content in zeolites; however, considering that all of the materials were obtained from the synthesis pot of the same composition, the influence of the amount of iron on the formation of oligonuclear species can be excluded in our case. According to Pérez-Ramírez and co-workers [75], oligonuclear moieties appear after the removal of the template during calcination. The authors postulated that iron–framework bonds are broken at high temperatures, which results in the dislodgement of Fe^3+^ into extraframework positions or even agglomeration of the metal species. However, the FeM22 structure is free from (Fe_x_^3+^O_y_) clusters, despite the fact that the sample was calcined at a higher temperature. Therefore, the formation of oligonuclear species can be correlated with the 2D, delaminated structure of M36 and I2. First of all, the interlayer distance of FeM22 is shorter than that of FeM36; thus, the free space between the layers is very likely to facilitate the formation of more aggregated clusters on the external surfaces. Furthermore, in the case of delaminated FeI2, the presence of oligonuclear species can be explained by their deposition on the disorganized layers of the material. Alternatively, the acidic medium applied during the synthesis could partially extract iron species from the zeolitic framework, resulting in the diversification of its final form. In fact, it was shown that apart from isolated cations, iron can be present in Fe-modified MCM-22 in other forms [5]; however, in the cited research only post-synthesis modifications, such as ion exchange, were reported. Wet impregnation of zeolites is usually performed under acidic conditions, thus, successful ion exchange is inhibited by the diffusion limits and hydrolysis of FeOOH species. According to the literature, the hydrolysis results in the formation of bigger particles of iron oxide [76]. Thus, one-pot synthesis is much more beneficial to obtain well-dispersed isolated active sites. Moreover, the bridging oxygen in [HO–Fe^3+^–O–Fe^3+^–OH]^2+^ species was reported to non-selectively oxidize NH_3_ to nitrogen during SCR process above 400 °C [76]. Hence, their presence is expected to directly influence the catalytic performance. Since the absorption lines in the region above 350 nm are flat, no extra framework metal oxide clusters were formed during hydrothermal aging of the synthesis gels. The results reported in the literature confirmed that bigger Fe_2_O_3_ particles can block the zeolite channels and pores, hindering the access of reacting molecules to the active site [76]. Moreover, this form of iron is inactive in SCR reaction [34] and similarly to Fe dimers, accelerates undesired ammonia oxidation above 400 °C [76]. The lack of bulk Fe_2_O_3_ particles on the external surface of the catalysts can be explained by a highly alkaline environment of the synthesis pot. Melian-Cabrera et al. [77,78] reported that in the case of Fe-ZSM-5, careful control of diffusion through the zeolitic channels results in hydrolysis of Fe^3+^(H_2_O)_6_ to gelatinous FeOOH. Its subsequent thermal decomposition yields iron oxide particles. Additionally, hydrolysis is a competitive process to the exchange or incorporation of Fe^3+^ into the zeolitic framework. According to the authors, diffusion time and length can be shortened by the application of strongly basic medium; therefore, due to the conditions of the synthesis procedure used in our study, the analyzed Fe-MWW zeolites are good candidates for the materials deprived of the external iron oxide species. The reported effect has a significant correlation with the catalytic performance of the materials.

### 2.2. Results of Catalytic Tests

#### 2.2.1. NO Conversion

**Figure 6:**
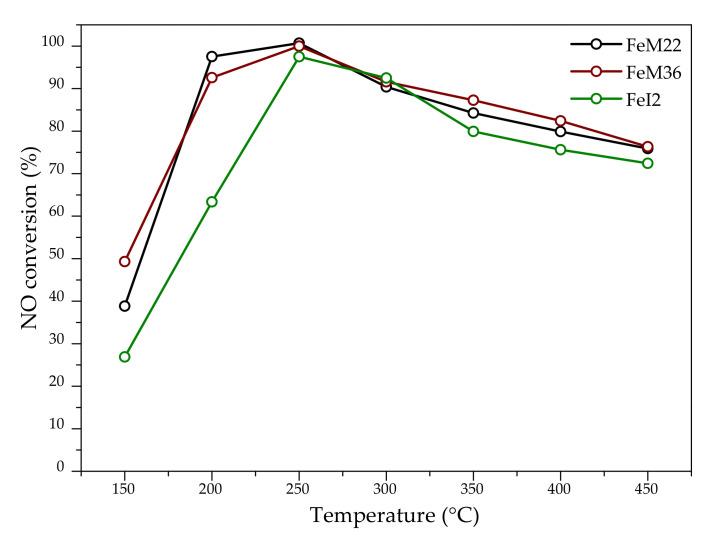
NO conversion obtained for the investigated one-pot synthesized Fe-MWW zeolites.

The results of NO conversion obtained for the catalysts are presented in Figure 6. It was proved that all of the tested materials are highly active in the examined temperature range of NH_3_-SCR. The catalysts exhibited NO conversion of almost 100% at 250 °C. It can be noticed that the activity of FeM22 and FeM36 oscillates around similar values, and 50% of NO conversion for the samples is reached at 160 and 150 °C (*t*_50_), respectively. Such a satisfactory result can be assigned to the abundance of isolated framework and extra framework Fe^3+^ species [34]. Thus, we validated the existing theory on the dependence of NH_3_-SCR activity on the type of metallic species. Gao et al. [72] performed a Mössbauer spectroscopy investigation over Fe-exchanged chabazite zeolites and suggested that the major active centers of NH_3_-SCR are extra framework monomeric [Fe(OH)_2_]^+^ and dimeric [HO–Fe–O–Fe–OH]^2+^. Høj et al. [58] reported a correlation between the amount of monomeric Fe sites and denitrification efficiency. The authors ascribed the promoting effect to the adsorption of NO on isolated Fe^3+^ moieties, which results in the formation of strong Fe–NO complexes, which is described by Reaction (R1) [72]:

[–Fe^3+^–O–] + NO → [–Fe^3+^–O–NO] → [–Fe^2+^–] + NO_2(ads)_ (R1)

However, according to the authors [72,79], the produced [–Fe^2+^–] cannot be oxidized by O_2_ or O_2_/NO/NH_3_ mixture during the reaction. On the other hand, DFT studies performed by Li and co-workers [80] proved that [FeOH]^+^ active center can be regenerated by NO_2_ and NH_3_. Taking into account the correlation between iron species and NH_3_-SCR activity, we can assume that the NH_3_-SCR reaction mechanism over Fe-MWW zeolites was promoted by the formation of NO_2_ produced by redox reaction on Fe^3+^ sites. The presence of NO_2_, indispensable for the fast NH_3_-SCR was confirmed to accelerate the reaction below 300 °C [72]. Additionally, according to some studies, oxidation of NO to NO_2_ is the rate-determining step of standard NH_3_-SCR. It was also confirmed that [FeOH]^+^ species are active in fast NH_3_-SCR at higher temperatures [81]; therefore, it can be assumed that NH_3_-SCR reaction over Fe-MWW zeolites follows the Mars–van Krevelen mechanism, assuming that Fe^3+^ is reduced to Fe^2+^ by NO, according to the Reaction (R2) [72]:

[–Fe^3+^–O–Fe^3+^–] + NO → [–Fe^2+^–O–Fe^2+^–] + NO_2(ads)_ (R2)

Reoxidation of the active center is then presented by the Reaction (3) [72]:

[–Fe^2+^–O–Fe^2+^–] + ½ O_2_ → [–Fe^3+^–O–Fe^3+^–] (R3)

However, based on our studies, it is not possible to confirm whether standard or fast NH_3_-SCR mode is dominant in the case of Fe-MWW zeolites. UV-Vis spectroscopy and operando EPR studies carried out by Vélez and co-workers [82] indicated that iron can be present in zeolites in three different positions, which behave differently under various conditions of the reaction. The monomeric iron sites in 10 MR (α positions) remained trivalent in standard and fast NH_3_-SCR. On the other hand, the sites in six MR (β sites) were reduced to inactive Fe^2+^ centers under standard conditions but were not affected during the fast mode of the reaction. Additionally, it is not clear if α or β iron sites are prevalent. On the one hand, after reduction, isolated cations in β positions are no longer active in standard NH_3_-SCR; however, due to the fact that the species can be reoxidized by NO_2_ [81]_,_ their catalytic activity is preserved in the fast NH_3_-SCR.

Interestingly, FeI2 showed high but noticeably lower NO conversion compared to other catalysts in the temperature range of 150–250 °C. The effect can be ascribed to the fact that the introduction of iron into the synthesis pot of ITQ-2 decreased the number of acidic centers compared to the pristine zeolite; therefore, the lower catalytic activity of Fe-ITQ-2 may have various reasons. Firstly, the strength of acidity of the bridged Me(OH)Si blocks (Me = Si, Al, Fe in the zeolitic framework) can be ordered as SiOH < Fe(OH)Si < Al(OH)Si [83]. Since delamination could promote the exfoliation of iron cations on the external surface of the delaminated layers, the material could contain the lowest amount of Fe framework species. Furthermore, the high-temperature peaks in NH_3_-TPD patterns are interpreted mainly as the Brönsted acidic sites [84]. In fact, these centers do not play a key role in NH_3_-SCR [85,86] but still are required to facilitate the uniform dispersion of metallic sites and prevent their undesirable agglomeration [76]. Additionally, they are responsible for the release of the adsorbed ammonia molecules at the high temperature of the reaction [81]. Moreover, Xu et al. [87] demonstrated that there is a synergistic effect between the isolated Fe^3+^ and the acidic centers of the zeolites. Another explanation of the decreased activity of FeI2 may have originated from the position of Fe sites in the zeolitic framework. Since ITQ-2 preserves only 10 MR inside the delaminated layers, it can be predicted that FeI2 suffered from a significantly lower amount of α Fe sites, which can be easily reoxidized during standard NH_3_-SCR. It must be noted, however, that the catalytic activity of the material above 250 °C was very close to the other studied zeolites; therefore, the formation of NO_2_, which regenerated iron sites in β positions and increased the reaction rate, cannot be excluded. In summary, in the case of Fe-ITQ-2, slightly lower NO conversion below 250 °C could be caused by the weaker acidity of the sample or the position of iron in the zeolitic structure. Following this conclusion, a significantly higher strong acidity of FeM22 and FeM36 contributed to the uniform dispersion of active sites and better catalytic performance of the materials below 300 °C.

Above 250 °C, the activity of the catalysts started to decrease gradually. This effect might be caused by the insufficient amount of oligomeric Fe sites, confirmed by UV-Vis studies. Kröcher and Brandenberger [55] analyzed the correlation between the speciation of the active iron sites in ZSM-5 and the behavior of the catalyst in NH_3_-SCR. In their carefully designed study, the authors showed that all forms of iron species participate in NO reduction during the catalytic process; however, this participation strongly depends on the reaction temperature: the monomeric active species delivered denitrification activity at the lowest temperature (<300 °C), (Fe_x_^3+^O_y_) oligomers were important active sites for the reaction above 300 °C, while Fe_2_O_3_ nanoclusters contributed to NO conversion at the temperature exceeding 450 °C. In our study, we confirmed that the majority of iron species are represented by the isolated framework and extra framework cations. Based on the activity order presented by Kröcher and Brandenberger, we confirmed that the correlation also exists for Fe-MWW materials; therefore, the slightly decreasing activity above 300 °C may result from the low diversity of different forms of iron.

#### 2.2.2. N_2_O Concentration

Nitrous oxide is one of the side products of the NH_3_-SCR reactions, which significantly limits the selectivity of the catalyst to N_2_. Moreover, the formation of N_2_O is highly undesired due to its strong greenhouse character [88]; therefore, its emission from the industrial NH_3_-SCR units has to be strictly controlled. In general, the formation of N_2_O during the reduction of NO with ammonia can take place according to Reactions (R4)–(R8):

2 NH_3_ + 2 NO_2_ → N_2_O + N_2_ + 3 H_2_O (R4)

3 NH_3_ + 4 NO_2_ → 3.5 N_2_O + 4.5 H_2_O (R5)

2 NH_3_ + 2 O_2_ → N_2_O + 3 H_2_O (R6)

4 NH_3_ + 4 NO_2_ + O_2_ → 4 N_2_O + 6 H_2_O (R7)

4 NH_3_ + 4 NO + 3 O_2_ → 4 N_2_O + 6 H_2_O (R8)

However, one should note that, under typical NH_3_-SCR conditions, Reactions (R6) and (R8) have not been observed for metal-exchange zeolites [76].

**Figure 7:**
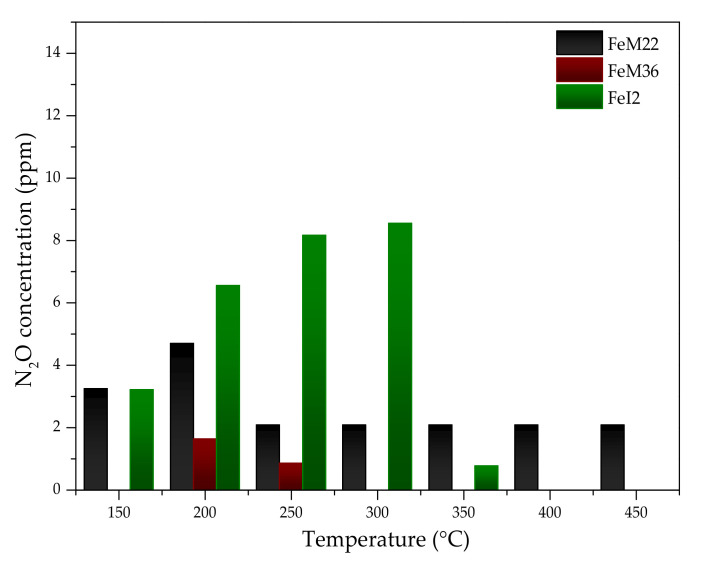
Concentration of N_2_O in the gas mixture during NH_3_-SCR catalytic tests conducted over Fe-MWW zeolites.

The concentration of N_2_O in the gas mixture during NH_3_-SCR experiments over Fe-MWW zeolites is presented in Figure 7. The amount of N_2_O did not exceed 10 ppm for any of the analyzed catalysts, and the value is within the experimental error of the used analyzer. In the case of FeM22, the concentration of N_2_O was similar (2–5 ppm) in the whole temperature range, while for FeM36, it was practically below the detection level. On the contrary, FeI2 exhibited a higher N_2_O concentration of 3–9 ppm. The emission of nitrous oxide for this sample showed an increasing trend within 150–300 °C, while above 350 °C N_2_O was almost absent in the post-reaction gas mixture.

The formation of nitrous oxide during the reaction over FeM22 and FeI2 zeolites can be correlated with the predicted presence of NO_2_ in the reacting gas. Devadas and co-workers [89] reported that, in a low-temperature range, low amounts of nitrogen dioxide can lead to slight emissions of N_2_O. The mechanism of the reaction was explained in detail by Gao et al. [72]. The authors postulated that NO is firstly oxidized during NH_3_-SCR and a part of the produced NO_2_ is immediately adsorbed on the catalyst surface. NO_3_^−^ species produced during the chain reaction are expected to be the precursors of NH_4_NO_3_ deposits formed at low temperatures. Above 200 °C, the deposits are decomposed according to Reaction (R9):

NH_4_NO_3_ → N_2_O + 2 H_2_O (R9)

This conclusion was supported by Grossale et al. [90], who also declared that N_2_O in Fe-zeolite based-systems is produced mainly during the thermal decomposition of ammonium nitrate. Additionally, it was reported that the rising temperature increases the oxidation activity of metal-exchanged zeolites [91]. Thus, negligible amounts of N_2_O for FeM22 in the whole temperature range can be explained by the occurrence of Reactions (R4), (R5), and (R7). The highest concentration of N_2_O obtained for FeI2 can result from the highest oxidizing properties of the material, caused by the specific position of iron sites in the sample and different arrangement of layers, in comparison to FeM22 and FeM36; however, it can be expected that above 350 °C, the catalyst is active in the decomposition of N_2_O to nitrogen and oxygen, according to Reaction (R10):

2 N_2_O → 2 N_2_ + O_2_ (R10)

Nevertheless, the temperature is lower than that of the standard scope for N_2_O decomposition over iron-based catalysts [92]; therefore, more detailed studies are required in order to confirm the activity of FeI2 in this reaction.

## 3. Materials and Methods

### 3.1. Preparation of the Materials

In order to prepare MCM-22 (P), we followed the procedure reported by Corma et al. [93]. The precursor was hydrothermally synthesized from a mixture with the following molar composition: SiO_2_: 0.02 Al_2_O_3_: 0_._5 HMI: 0.09 NaOH: 45 H_2_O. Firstly, 0.375 g of NaOH (MiliporeSigma, Burlington, MA, USA) and 0.375 g of NaAl_2_O_3_ (56% Al_2_O_3_, 37% Na_2_O) were dissolved in 81.71 g of Mili-Q water (Merck, Darmstadt, Germany) and stirred for 5 min at room temperature. Subsequently, 6 g of silica (Aerosil 200, Degussa, Frankfurt, Germany) was added slowly to the mixture, maintaining constant stirring. Last but not least, 4.96 g of hexamethyleneimine (HMI, 98 wt.%) was dropwise introduced to the solution and the resulting gel was mixed for 2 h. Finally, the synthesis gel was crystallized in a Teflon-lined, stainless-steel autoclave under rotation (60 rpm) at 150 °C for 7 days. The obtained solid was filtered, washed with distilled water to neutral pH, and dried overnight at 100 °C.

**Figure 8:**
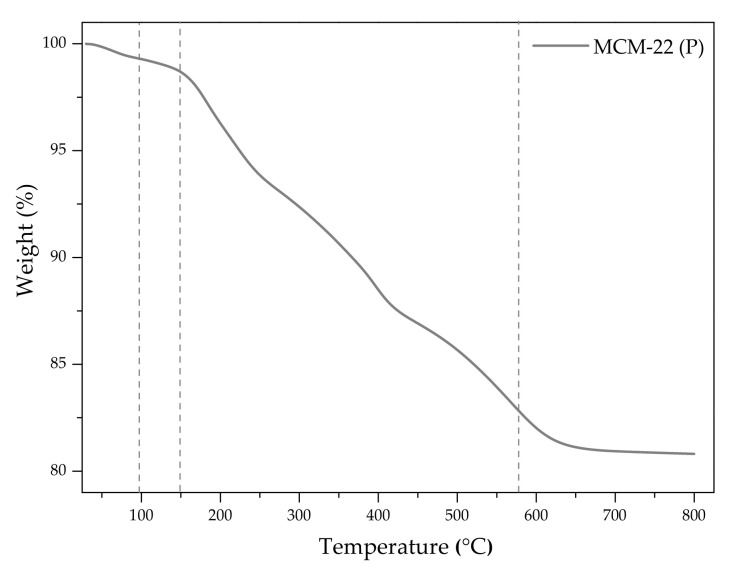
TGA profile of the precursor MCM-22 (P) obtained in the temperature range of 30–800 °C.

The preparation of MCM-22 was based on the thermal treatment of the precursor under appropriate conditions. Thus, MCM-22 (P) was calcined in the following temperature ramps: 100 °C for 2 h, 150 °C for 2.5 h, 350 °C for 3 h, and 580 °C for 3 h. As demonstrated in Figure 8, which presents the results of TGA of the precursor, the gradual elimination of the organic template started at 100 °C and finished around 600 °C.

The temperature ramps applied between 100 and 580 °C provided gradual removal of the organic template from the precursor without the formation of coke or steam effects, which could modify the structure and/or the internal porosity of the zeolite. Additionally, long and progressive calcination favors better preservation of the integrity of the zeolitic framework. The precursor weight decreased by ca. 20% during calcination. The obtained material was labeled as M22.

The method of the synthesis of MCM-36 used in the following study was adapted from that described by Jankowska et al. [10]. In order to prepare the pillared micro-mesoporous zeolite, the sheets of MCM-22 (P) were expanded (“swollen”) by the introduction of the organic molecules in the interlayer space. The swelling solution of centyltrimetyulammonium bromide (CTMA^+^Br^−^) and tetrapropylammonium bromide (TPA^+^Br^−^), in which the bromide ions were partially exchanged (70% in the case of CTMA and 30% in the case of TPA) for hydroxide anions, using a hydroxide form of strong anion exchange resin (Amberlite IRN78). The swelling procedure consisted of the following steps: 5 g of MCM-22 (P) was dispersed in the solution of 100 g of CTMA^+^Br^−^/OH^−^ and 30 g of TPA^+^Br^−^/OH^−^ and Mili-Q water. The mixture was stirred under reflux at 80 °C for 16 h. Afterwards, it was filtered, washed several times with distilled water to neutral pH, and dried overnight at 60 °C in air. SiO_2_ pillars were introduced into the interlayer space of the swollen material using tetraethyl orthosilicate (TEOS). The weight ratio of the solid to the pillaring solution was 1:5; the suspension was stirred at 80 °C for 24 h in N_2_ atmosphere. Subsequently, the mixture was filtered and the solid material was washed and dried overnight at 60 °C in air. The obtained product was hydrolyzed in distilled water at pH ca. 9.0, optimized by 25% aqueous solution of NH_3_·H_2_O. The hydrolysis was carried out at 40 °C for 6 h. Finally, the sample was filtered and dried overnight at 60 °C in air. The resulting solid was calcined at 540 °C for 1 h in N_2_ atmosphere and for 6 h in air. The material was labelled as M36.

For the synthesis of ITQ-2 zeolite, we followed the method proposed by Corma and co-workers [94]. Typically, the swollen precursor, prepared analogically to that of MCM-36, was introduced into the ultrasound bath (50 W, 40 kHz) for 1 h. Subsequently, the solution was acidified by 1 M hydrochloric acid until pH of the slurry was below 2. Afterwards, the formed solid was centrifuged. The organic molecules of the residual swelling solution were removed by calcination at 540 °C for 1 h in N_2_ atmosphere and for 6 h in air. The material was labeled as I2.

In order to prepare Fe-zeolites by one-pot synthesis, we adopted the procedure reported by Chen et al. [16]. The molar composition of the reacting gel was SiO_2_: 0.017 Al_2_O_3_: 0.05 Fe(NO_3_)_3_: 0.5 HMI: 0.4 NaOH: 45 H_2_O, which gives Si/Al and Si/Fe molar ratio of 30 and 20, respectively. The appropriate amounts of NaOH, NaAlO_2,_ and Fe(NO_3_)_3_·9 H_2_O were dissolved in deionized water and stirred for 10 min at room temperature. Subsequently, the silica sol (40.5 wt.% of SiO_2_) and HMI were added dropwise into the gel. The as-prepared mixture was vigorously stirred for 2 h, transferred into Teflon-lined stainless-steel autoclaves and left for crystallization under rotation mode at 150 °C for 7 days. The obtained solid was filtered, washed properly with distilled water to pH 7 and dried at 100 °C for 24 h. The resulting precursor, Fe-MCM-22 (P), was modified analogically to the pristine materials in order to obtain Fe-MWW zeolites. The as-prepared catalysts with iron were labeled as FeM22, FeM36, and FeI2.

### 3.2. Characterization of the Materials

The chemical composition of the catalysts (Si, Al, Fe content) was analyzed using inductively coupled plasma optical mass spectroscopy (ICP-OES, QTEGRA).

In order to identify the crystallinity and phase purity of the layered zeolites and the catalysts, X-ray powder diffraction (XRD) was applied. The XRD patterns were obtained using an Empyrean (PANalytical) diffractometer (PANalytical, Almelo, UK) equipped with a Cu-Kα radiation source (λ = 1.54184 Å) at a tube current of 40 mA and a voltage of 40 kV. The scanning range of *2θ* was set for 2–40 ° or 3–90 °, with a scan step of 0.02 ° and counting time of 1 s per step. Data analysis was performed with the software X’pert HighScore software plus (with database) (Malvern Panalytical, Malvern, UK).

The nitrogen adsorption–desorption isotherms were measured at 77 K by Micromeritics gas adsorption analyzed and 3Flex Surface Characterization software. Prior to the analysis, the samples were degassed under vacuum: at 90 °C for 1 h and at 350 °C for 5 h. The specific surface area (*S_BET_*) of the materials was calculated using the *BET* (Brauner–Emmet–Teller) model, from the adsorption branch, according to the recommendations of Roquerol [95]. Taking into consideration the specific shape of the isotherms pointing at the micro-mesoporous structure of the catalysts, the external surface area, surface of micro- and mesopores, and their volume was calculated using the t-plot method.

The concentration and strength of the acid sites present in the catalysts were determined by temperature-programmed desorption of NH_3_ using an Autochem II (Micrometrics) apparatus. The experiments were performed in the temperature range of 100–600 °C in a fixed bed continuous flow microreactor. Prior to each measurement, 150 mg of each sample was treated with a stream of argon at 100 °C for 60 min. Afterwards, each of the analyzed materials was equilibrated at 100 °C with a stream of helium and saturated for about 30 min in a flow of 1 vol.% of NH_3_ in He. Subsequently, the analyzed catalyst was heated gradually with a ramp of 10 °C·min^−1^, up to 600 °C in an argon stream. The desorbed amount of ammonia was analyzed by means of a thermal conductivity detector (TCD) and coupled GC-MS mass spectrometer (OmniStar, Bazers Instruments).

The characteristic chemical groups of the layered zeolites framework and the catalysts were studied by Fourier-transform-infrared spectroscopy (FT-IR). The spectra were collected with a Perkin Elmer Frontier spectrometer in the wavelength region of 4000–400 cm^−1^, with a resolution of 4 cm^−1^. Before the measurement, each sample was mixed with KBr with the ratio of 1:100 and pressed into the disk.

The speciation and distribution of iron species introduced into the zeolite framework were determined by ultraviolet diffuse reflectance spectra (UV-vis-DR). The analysis was carried out with a Cary 5 spectrophotometer equipped with a diffuse reflectance accessory. The spectra were taken in the range of 200–900 nm, with a resolution of 2 nm.

### 3.3. NH_3_-SCR Catalytic Tests

NH_3_-SCR catalytic tests over the prepared catalysts were conducted in a fixed-bed flow microreactor with a quartz tube under atmospheric pressure. Firstly, 0.2 g of the sample was outgassed in a flow of nitrogen at 400 °C for 30 min. After cooling down to 100 °C, the material was exposed to the model gas mixture containing 800 ppm of NO, 800 ppm of NH_3_, 3.5 vol% of O_2_, and He as an inert. The total gas flow was 100 cm^3^·min^−1^. The measurements were carried out in the temperature range of 150–450 °C with 50 °C ramps. NO_2_ formed during the reaction was decomposed to NO by the catalytic converter downstream of the microreactor. The concentrations of residual NO and N_2_O (the by-product of the reaction) in the outlet gas were analyzed continuously by the FT-IR detector (ABB 2000, AO series). In order to calculate NO conversion, the formula represented by Equation (1) was used:

(1) $$\mathrm{NO}\;\mathrm{conversion}\;(\%)=\frac{C_{\mathrm{NO}\left(\mathrm{in}\right)}-C_{\mathrm{NO}\left(\mathrm{out}\right)}}{C_{\mathrm{NO}\left(\mathrm{in}\right)}}\;\times \;100\%$$

where C_NO(in)_—inlet concentration of NO, C_NO(out)_—outlet concentration of NO in the gas mixture.

## 4. Conclusions

Our study has shown that one-pot synthesis advantageously influenced the catalytic activity of Fe-MWW zeolites in NH_3_-SCR. The introduction of the iron precursor into the synthesis pot only slightly changed the surface properties of the materials, as the consequence of the isomorphous substitution of Fe^3+^ and Si^4+^ or Al^3+^ in the zeolitic framework. The reported results confirmed that the introduction of Fe promoted the formation of new, strong acid sites. The majority of iron species were present in the form of well-dispersed, isolated cations, which enhanced NO conversion below 250 °C. Furthermore, the presence of oligomeric moieties was responsible for high activity in the medium temperature region. The gradually decreasing reduction of nitrogen oxide above 250 °C, observed for all catalysts, possibly resulted from the low diversity of the types of iron species; however, good dispersion of the active phase provided negligible production of N_2_O during the reaction. Our findings indicated that the arrangement of MWW layers determined the type of acidity and speciation of the introduced metal, thus, catalytic performance of the investigated materials. Moderately lower activity of FeI2 sample in the low-temperature region and higher N_2_O production was a consequence of pore blockage by more aggregated Fe_x_O_y_ clusters, hence, the possible occurrence of side reactions. In summary, our study has highlighted the positive impact of one-pot synthesis on the isomorphous incorporation of the catalytically active phase into zeolitic frameworks. The presented work can be beneficial for the design of new catalysts, which require a well-dispersed form of active centers.

## Figures and Tables

**Table 1:** The content of Si, Al, and Fe with the corresponding Si/Al and Si/Fe molar ratios in the non-non modified zeolites and the catalysts.

Sample Code	Si (wt.%)	Al (wt.%)	Fe (wt.%)	Si/Al	Si/Fe
M22	33.23	1.41	-	25	-
M36	33.98	1.16	-	28	-
I2	41.02	1.46	-	27	-
FeM22	36.58	1.17	4.78	29	15
FeM36	34.02	1.16	5.02	28	13
FeI2	36.38	1.17	4.78	29	15

**Table 2:** Textural and structural properties of the samples recognized from N_2_ sorption experiments.

Sample Code	S_BET_^a^ (m^2^·g^−1^)	S_micro_^b^ (m^2^·g^−1^)	S_ext_^b^ (m^2^·g^−1^)	V_total_^c^ (cm^3^·g^−1^)	V_micro_^b^ (cm^3^·g^−1^)	V_meso+macro_^d^ (cm^3^·g^−1^)
M22	590	338	158	0.431	0.169	0.262
M36	569	434	141	0.480	0.172	0.308
I2	539	375	164	0.450	0.183	0.267
FeM22	438	381	77	0.482	0.185	0.297
FeM36	716	209	507	0.740	0.162	0.578
FeI2	550	350	201	0.635	0.174	0.461

^a^ Surface area determined by BET method; ^b^ Micropore surface area, external surface area, and micropore volume determined by t-plot; ^c^ Total pore volume at p/p_0_ = 0.98 cm^3^·g^−1; d^ V_micro+meso_ = V_total_ − V_micro__._

**Table 3:** Quantitative evaluation of acid centers of the pristine and Fe-modified MWW zeolites.

Sample Code	Concentration of Acid Sites (μmol·g^−1^)		
	Weak Sites	Strong Sites	Total Amount of Sites
M22	761	535	1296
M36	774	596	1370
I2	747	504	1251
FeM22	1158	813	1971
FeM36	580	427	1007
FeI2	546	421	967

## Data Availability

Data are contained within the article.

## Sample Availability

Catalyst samples are available at AGH University of Science and Technology.
